# Hospitalizations during the last months of life of nursing home residents: a retrospective cohort study from Germany

**DOI:** 10.1186/1472-6963-6-70

**Published:** 2006-06-08

**Authors:** Heribert Ramroth, Norbert Specht-Leible, Hans-Helmut König, Hermann Brenner

**Affiliations:** 1 German Centre for Research on Ageing, Heidelberg, Germany; 2 Department of Tropical Hygiene and Public Health, Heidelberg, Germany; 3 Bethanien-Hospital, Geriatric Centre at the University Hospital, Heidelberg, Germany; 4 Health Economics Research Unit, Department of Psychiatry, University of Leipzig, Leipzig, Germany; 5 Department of Clinical Epidemiology and Research on Ageing, German Cancer Research Center, Heidelberg, Germany

## Abstract

**Background:**

To describe hospitalisations of nursing home (NH) residents in Germany during their last months of life.

**Methods:**

Retrospective cohort study on 792 NH residents in the Rhine-Neckar region in South-West Germany, newly institutionalized in the year 2000, who died until the study end (December 2001). Baseline variables were derived from a standardized medical examination routinely conducted by the medical service of the health care insurance plans in Germany. Information on hospitalisations and deaths was extracted form records of the pertinent health insurance plans.

**Results:**

NH residents who died after NH stay of more than 1 year spent 5.8% of their last year of life in hospitals. Relative time spent in hospitals increased from 5.2% twelve months before death (N = 139 persons) to 24.1% in their last week of life (N = 769 persons). No major differences could be observed concerning age, gender or duration of stay in NH. Overall, 229 persons (28.9%) died in hospital. Among these, the last hospital stay lasted less than 3 days for 76 persons (31.9%). Another 25 persons (3.2%) died within three days after hospital discharge.

**Conclusion:**

Our study indicates that proximity of death is the most important driver of health care utilization among NH residents. The relation of age or gender to health care expenditures seem to be weak once time to death is controlled for. Duration of NH stay does not markedly change rates of hospitalisation during the last months of life.

## Background

The present foreseeable demographic development in Germany shows a strong increase in the proportion of older people in the total population, which is even stronger than in most other developed countries [1]. Due to the reduced mortality rate, a very dynamic development will take place in the higher age groups, especially the oldest old. In 2050 one third (36.7%) of the German population will be 60 years and older, 12.1% will be more than 80 years old (2001: 24.1% and 3.9% respectively).

Older persons are more dependent on medical and nursing care facilities than other population groups. Among all persons 75 years and older in Germany 23% were dependent on permanent care (in 2001), for persons 90 years and older the probability for permanent care dependence was 60%, with a much higher percentage in women (65%) than in men (42%) [2]. Nursing home residents represent a group with a particularly high demand on medical and nursing care. In 2001, two million Germans have been in need of nursing care and 604,000 persons have been institutionalized in 9,165 nursing homes (NHs), with a decreasing trend for family care and an increasing trend for institutional care at the end of life [3].

Recent studies indicate that approaching death, rather than age, may be the main demographic driver of health care utilization and costs [4-6]. Due to the concentration of expenditures at the end of life, provision of adequate medical care for frail disabled older persons is of particular relevance for the health care system. Despite limited financial resources the special needs of care dependent old persons must be met.

In Germany, a relatively comprehensive system of social insurance has been built in the past, with nursing care insurance being responsible for nursing care, and health insurance for medical care. NHs serve personal care needs (support in the activities of daily living) and specialized nursing needs (e.g. wound care). They do not provide primary health care, which is the domain of general practitioners in Germany. Nearly all cases of acute illness are referred to acute hospitals, in contrast to other countries such as the United States and Great Britain, where (sub-)acute hospital care is often provided by NHs. Therefore, the relative contributions of NHs and acute care hospitals must be well defined.

In the United States the relationship between NHs for older persons and hospitals has been studied over years [7-11]. By contrast, little is known about hospitalisations and their determinants among NH residents during their last months of life in European countries.

The aim of this study was to assess rates of hospitalisations and their determinants among NH residents in Germany before and after nursing home admission (NHA). A first comparison of the rates before and after NHA have been presented elsewhere [12]. The present publication shows the rates during the last months of life of German NH residents.

## Methods

### Study design and study population

**Figure 1:**
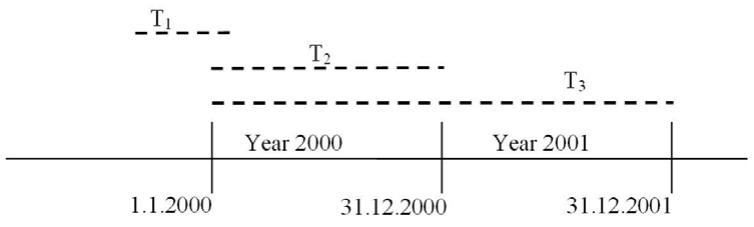
Study design: Time windows for standardized medical examination (T1), Nursing home admission (T2) and death (T3).

A retrospective cohort study was set up in the cities of Heidelberg, Mannheim and the Rhine-Neckar area, a region with 829.930 inhabitants. The study population included 1926 NH residents who were newly institutionalized between 1^st ^of January and 31^st ^of December 2000 in one of the 97 NHs in the study region who applied for benefits from the German statutory nursing insurance system (see Figure 1). According to statutory regulations, benefits from the German statutory nursing insurance are provided to those people who need support in the activities of daily living (ADLs) (personal hygiene, nutrition and mobility one or more times a day) because of severe functional or cognitive impairment. Frequency and duration of support determine the level of care benefits. About 98% of all NH residents in Germany receive such benefits [3]. The benefits are granted contingent upon the results of a standardized medical examination carried out by the medical service of the health insurance plans. In this retrospective cohort study, we linked sociodemographic data as well as data of these medical examinations with follow-up data regarding hospitalisations from the health insurance plans. For logistic reasons, the sample was restricted to members in either one of the eight largest health insurance plans, which cover about 80% of the population.

During the study period 792 participants died. In this analysis, we will refer to this sub-cohort of deceased persons.

The study was entirely based on administrative and anonymously analysed data by the medical service of the health care insurance in Germany and of the pertinent health insurance plans and was approved by the Ethics committee of the University of Heidelberg and by the responsible state commissioner of data protection.

### Baseline examination

The standardized medical examinations by the medical service of the health care insurance plans consist of a physical examination as well as a detailed interview (using the help of relatives or care givers if necessary) and careful observation of the applicant. They are carried out by trained physicians according to written guidelines that describe all medical, functional, and social aspects that need to be taken into account. The final decision for qualifying for the benefits is made by a physician, taking into account routine data, but also specific individual particularities of the applicant. Re-examinations are carried out in case of further applications for a higher level of benefits from the nursing care insurance, in case of changes in care status, but not in every case of NH admission (NHA). Thus the date of this examination is not in every case very close to the date of NHA. Nevertheless nearly 60% (N = 459) of the examinations took place within three months before or after nursing home admission, another 20% (N = 157) within 1 year around NHA, whereas 95 (12%) examinations were more than 1 year away from NHA. In 10% of all records, the date of baseline examination was missing.

Medical diagnoses describing the underlying medical causes of dependency on permanent care are documented in rank order according to their relevance for impairment in the standardized medical examinations. For this analysis, the main medical cause (i.e., the diagnosis ranked first by the physician) was coded according to the International Classifications of Diseases 9 and 10 (Table 1).

The medical examination provided detailed information on the level of dependency and time of care required regarding basic activities of daily living. In 10% of these medical examinations only the total time for need of help was given. Therefore the level of care dependency was divided in quartiles of this total time variable for this analysis.

Age was determined at the time when entering the NH.

### Follow-up variables

Longitudinal information on hospitalisations and deaths until 31^st ^of December 2001 was extracted from records of the pertinent health insurance plans. Admissions to acute-care hospitals with length of stay during the follow-up period were registered (see Figure 1).

In addition to the dates of hospital entry and discharge, the leading diagnosis for hospitalisation as given by the acute care hospital at discharge, as well as hospitalisation costs were extracted from the records. The discharge diagnoses were classified according to the International Classifications of Diseases 9 and 10.

### Statistical methods

Individual person-times at risk were calculated from the date of nursing home admission to the end of life, subtracting the time spent in hospital.

Relative time spent in hospital (RT), absolute rates of hospitalisations (HR) defined as the number of hospitalisations per person-year at risk, mean duration (MD) of hospital stay, and person-years (PY) in hospital and total person-years under observation were calculated according to age, gender, level of care dependency and time spent in NH. Presentation of results focuses on the relative time spent in hospital, combining the information of hospitalisation rates and mean duration of hospital stay. For all analyses the statistical software SAS (Version 8.2) was used.

For two of the largest health insurance plans data about hospitalisations before NHA was available for a sub-cohort of 559 deceased persons. Thus, for this sub-cohort the transfer status into NH from hospital or home could also be considered.

## Results

### Sample characteristics

Major sample characteristics are shown in table 1. The gender distribution reflects the considerably higher proportion of women (N = 560, 70.7%, mean age 84.7 years) among the older population caused by differences in life expectancy (men: N = 232, 29.3%, mean age 79.3 years). More than 74% of the study participants (men 58.2%, women 81.8%) were older than 80 years. The leading medical diagnoses for dependency on permanent care were dementia (N = 131, 19.0%), cerebrovascular diseases (N = 92, 13.3%) and cancer (N = 90, 13.0%: men 18.5%, women 10.7%). Three quarters of the participants (74.1%) were in need of nursing care for more than 1 hour per day at the time of the standardized medical examination. Survival time for 139 participants was more than 1 year. According to the definition of the observation window (the last year of life), the observation period for these 139 so called long-term NH residents was exactly 365 days. For the remaining 653 participants the mean observation period from NH admission to death was 120.9 days. Two hundred and eighty-nine persons (51.7%) had been transferred directly from acute care hospital to the NH.

### The last months of life

In the first month after NHA, 168 (21.2%) residents died and another 295 (37.2%) died during the next 5 months. 229 persons (28.9%) died in hospital. Among these, the last hospital stay lasted less than 3 days for 76 persons (9.6% of total sample). Another 25 (3.2%) persons died within 3 days after hospital discharge.

Nearly 58% (N = 459) of 792 study participants had at least one hospitalisation during their last months of life. In a total person-time of 355.2 person-years under observation a total of 849 transfers to acute hospitals have been observed.

**Figure 2:**
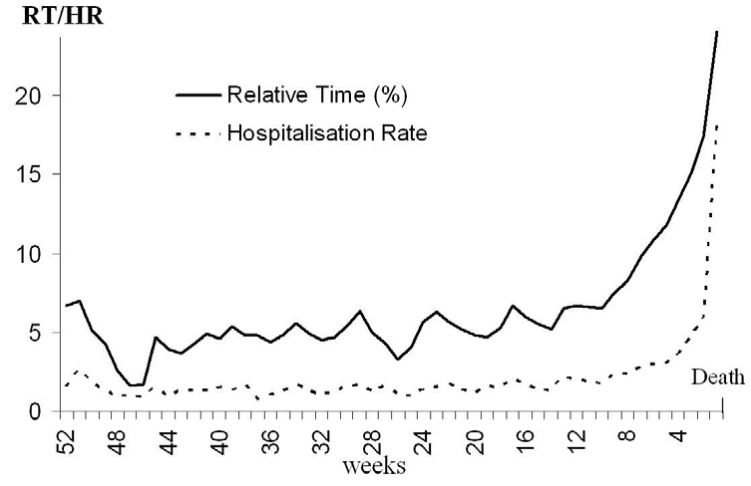
Relative time spent in hospital and hospitalisation rate during the last months of life (weekly rates).

Onehundredthirtynine NH residents have been followed for their whole last year of life. They spent 5.8% of this last year of life in hospital, their mean hospitalisation rate was 1.9 per person-year with a mean duration of 11.7 days per hospitalisation. Hospitalisation rates and time spent in hospital strongly increased as death approached: starting from 5.2% at twelve months before death RT increased up to 24.1% during the last week of life (Figure 2). Likewise, HR increased from 1.8 to 18.1 hospitalisations per person-year. No major differences could be observed concerning age, gender or duration of stay in NH. If anything, hospitalisation rates for people aged 90 years and older were lower than for the other age groups (see Table 2).

No clear relationship was found between dependency on level of care and the rates of hospitalisation. If anything, a weak inverse trend was seen (namely the higher the dependence on permanent care, the lower the relative time spent in hospital), which persisted after controlling for age in additional analyses.

The rates for people who could be observed for more than one year after NHA increased in the same manner and dimension as for residents with short NH stay (Table 2).

The leading causes of hospitalisations during the last months of life (Table 3) were cardiovascular diseases (N = 146, 17.2%), infections (N = 104, 12.2%), diseases of the digestive system (N = 93, 11.0%) and injuries (N = 78, 9.2%). Pneumonia and influenza were the cause for hospitalisations in more than half of the group of infections N = 62 (7.3%). Cardiovascular diseases accounted also for the largest share of time spent in hospital (15.6%), followed by infections (12.6%) and psychiatric diseases (11.4%).

The leading causes of costs during the last months of life were nearly the same as the leading causes of hospitalisations, namely cardiovascular diseases (15.6%), infections (12.9%) and in the same ranking injuries (10.4%) and diseases of the digestive system (10.0%).

Main diagnoses of last hospital stay for those who died in hospital were cardiovascular diseases (N = 48, 21.0%), diseases of the respiratory system (N = 41, 17.9%) and infections (N = 31, 13.5%). The same rank order and nearly the same proportions were found for the last hospitalisation of those who died within 3 days after discharge.

## Discussion

A cohort of 792 persons who applied for benefits from the German statutory nursing insurance system and who were newly admitted to a NH in the year 2000 was retrospectively observed from NHA to death. To our knowledge, this was the first European longitudinal study regarding hospitalisation rates in the last months of life in a population-based sample of NH residents.

NH residents who died after NH stay of more than 1 year spent 5.8% of their last year of life in hospitals. Relative time spent in hospitals increased from 5.2% twelve months before death (N = 139 persons) up to one quarter of time in hospital during the last week of life (N = 769 persons). No major differences in hospitalisation rates during the last months of life could be observed concerning age, gender and duration of stay in NH. However, for all observed subgroups rates increased strongly during the last weeks of life.

The findings of increasing hospital rates before death are consistent with results of longitudinal studies from the US [13-15]. Along with the absence of major differences of hospitalisation rates according to age this pattern underlines findings from other studies, that health care costs are more directly related to proximity to death than to age [5,16,17].

Like in a previous study among handicapped community-dwelling older people from Germany [18], there was no clear relationship between level of care dependency and the rates of hospitalisation. If anything, a weak inverse trend was seen (namely the higher the dependence on permanent care, the lower the mean duration of stay per hospital transfer, and the relative time spent in hospital), which persisted after control for age in multivariate analyses.

During the last months of life, no major differences could be observed between the rates of those residents who stayed in NH for more than one year, and those who had lived in NH for less than one year, which underlines further the importance of proximity to death as the key trigger of increased hospitalisation rates.

Overall, the hospitalization rates of deceased NH residents found in our study are much higher than corresponding rates from the United States. Besides the differences in health care delivery and payment systems, lower rates in the US may partly be explained by wide spread use of do-not-resuscitate (DNR) orders [19], although DNR-orders do not directly deal with hospitalization. Culberson et al. (2005) saw a small effect of 'Do-not-hospitalize' (DNH)-orders, which are though quite less frequent than (DNR)-orders in the US. Both, DNR- and DNH-orders are still quite uncommon in Germany. According to Teno (2004), subconscious benign neglect is another more likely explanation for lower hospitalisation rates in the United States.

Comparing our results with those of Bickel (1998) who reported an overall time spent in hospital during the last year of life of 9.3% in a representative population study of deceased persons in the same region of Germany, the relative time spent in hospital by deceased NH residents may appear to be lower than in the general population. Additionally, death in the general population in the study of Bickel occurred in hospitals in about half of the cases (49.7%), a proportion which is much higher than in our study (29.7%). The reasons for the lower proportion of time spent in hospital during the last months of life of deceased NH residents compared to the general population might reflect a trend to deal more cautiously with hospital transfers of frail old people and to avoid high-technology interventions [7,18] or 'risky' treatments with uncertain benefit in the oldest old [20,21]. Additionally, the lower rates for the oldest old (aged 90 years and older) underline findings in the general German population that with advancing age, the utilization of NHs and ambulatory services rise steeply, whereas the probability of hospital treatment decreases [22,23].

There is a widespread discussion about further reduction of hospitalisation rates of NH residents during their last months of life. In a study from the United Kingdom, Snape et al. (1998) found that some substantial proportion of hospitalisations of persons who died within 3 days in hospital were inappropriate. In contrast, Stooker et al. (2001) conclude, that interventions to reduce costs of hospitalisations during the last year of life will have only a modest impact compared to the total health care budget. Hospitalisations of NH residents might possibly be further reduced by enhanced training of NH staff. In our study, injuries, infections, cardiovascular diseases and psychiatric diseases were the leading causes of hospitalisation. Some intervention trials have suggested that measures to control or manage infections, closer monitoring of cardiovascular disease, prevention of falls and fractures and geronto-psychological interventions have the potential to reduce hospitalisations in NH residents [10,24-30]. Thus, there seems to be a potential for further reductions of hospitalisations by pertinent interventions.

Issues for further research could be to characterize newly admitted NH residents according to risk factors associated with hospitalisation or mortality (Flacker et al. 2003, Porock et al. 2005). Further research should also address the following topics: Are hospitalisation rates of NH residents different from those of other people, when proximity of death is taken into account? Can hospitalisations be reduced by NH providing (sub-)acute-hospital care, and would this be cost effective? Can extensive hospitalisations before NHA be reduced by improving coordination between acute care and long-term care sectors? Are there effective strategies for preventing illness that causes hospitalisations of NH residents?

In the interpretation of our data, the following limitations should be kept in mind. Our analysis is based on data collected during medical examinations that were not performed for the specific purpose of this study. Unfortunately we could not consistently classify variables for mental disorders and specific activities of daily living, because 1–2 years before the time window of the present study, the classification system for the medical baseline examination changed, and thus data-records were not consistent in these variables. However, we were able to quantify the dependency with respect to ADL according to a summary variable of the average time for need of care per day. It has been shown that this time variable is strongly correlated with the restrictions on various ADLs [31,32].

Notwithstanding these limitations, our study extends the scarce database on hospitalisations among older disabled persons in Europe. It will provide important baseline data for assessing the impact of major changes in the social security system which are currently implemented in Germany, including implementation of a DRG-based prospective payment system.

## Conclusion

Our study indicates that proximity of death is the most important driver of health care utilization among NH residents. The relation of age or gender to health care expenditures seem to be weak once time to death is controlled for. Duration of NH stay does not markedly change rates of hospitalisation during the last months of life.

## Abbreviations

NH – Nursing home

NHA – Nursing home admission

RT – Relative time spent in hospital

HR – Hospitalisation rates

## Competing interests

The author(s) declare that they have no competing interests.

## Authors' contributions

HB designed and supervised the study and participated in its coordination. HR coordinated the study, performed the statistical analysis and drafted the manuscript. HB, NSL and HHK made major contributions to revisions of the paper. All authors read and approved the final manuscript.

## Pre-publication history

The pre-publication history for this paper can be accessed here:



## Figures and Tables

**Table 1:** Distribution of Age, Main Medical Cause for Dependency on Nursing Care and Level of Care Dependency among nursing home residents who died during the follow up period

Factor	men	women	total
**Age (in years)**			
<70	41 (17.7)	23 (4.1)	64 (8.1)
70 – 79	56 (24.1)	79 (14.1)	135 (17.0)
80 – 89	97 (41.8)	307 (54.8)	404 (51.0)
90+	38 (16.4)	151 (27.0)	189 (23.9)
**Main medical cause for dependency on nursing care (ICD-10/ICD-9)**^§^			
Infections (A00–B99, J10–J18, L00–L08, N30, N39/001–139, 480–487, 595, 599, 680–686	2 (1.0)	4 (0.8)	6 (0.9)
Cancer (C00–D48/140–199, 210–239)	38 (18.5)	52 (10.7)	90 (13.0)
Hormone-, nutrition-, metabolic diseases (E00–E90, N18–N19/240–279, 585–586)	1 (0.5)	13 (2.7)	14 (2.0)
Psychiatric Diseases (F00–F99/290–319)	47 (22.9)	134 (27.6)	181 (26.2)
- among these: Dementia (F00–F03/290)	33 (16.1)	98 (20.2)	131 (19.0)
Diseases of the nervous and sensory system (G00–G44, G47–G99/320–359)	18 (8.8)	37 (7.6)	55 (8.0)
Cerebrovascular diseases (G45–G46, I60–I69/430–438)	29 (14.1)	63 (13.0)	92 (13.3)
Cardiovascular diseases (D50–D89, I00–I59, I70–I99/200–208, 280–289, 390–429, 439–459)	11 (5.4)	29 (6.0)	40 (5.8)
Diseases of the musculoskeletal system (M00–M99/710–739)	14 (6.8)	45 (9.3)	59 (8.6)
Injuries and poisoning (R55, S00–T98/780, 800–999)	7 (3.4)	21 (4.3)	28 (4.1)
Diseases of the respiratory system (J00–J09, J19–J99/460–479, 488–519)	5 (2.4)	8 (1.6)	13 (1.9)
Diseases of the digestive system (K00–K93/520–579)	3 (1.5)	3 (0.6)	6 (0.9)
Other diseases	30 (14.6)	76 (15.7)	106 (15.4)
**Survival time (Time after NHA until death)**			
<= 2 weeks	28 (12.1)	66 (11.8)	94 (11.9)
> 2 weeks – 4 weeks	26 (11.2)	49 (8.8)	75 (9.5)
> 1 month – 3 months	54 (23.3)	112 (20.0)	166 (21.0)
> 3 months – 6 months	34 (14.7)	96 (17.1)	130 (16.4)
> 6 months – 12 months	48 (20.7)	140 (25.0)	188 (23.7)
> 1 year	42 (18.1)	97 (17.3)	139 (17.6)

Total	232 (100)	560 (100)	792 (100)

^$^102 persons without classification caused by missing values

**Table 2:** Hospitalisations during the Last Months of Life

			12–4 months before Death			≤3 months before Death		
		N^%^	HR	MD	RT	HR	MD	RT

Age (in years)	<70	64	2.1	13.7	7.3	4.8	11.3	12.8
	70 – 79	135	1.8	12.6	6.0	5.4	11.3	14.4
	80 – 89	404	1.4	13.6	4.9	4.5	11.6	12.4
	90+	189	1.1	14.0	4.0	3.7	9.9	9.1
Gender	Men	232	1.6	12.0	5.0	5.0	10.5	12.6
	Women	560	1.3	14.2	4.9	4.2	11.4	11.6
Care level^$ ^(in minutes)	0 – <= 60	144	1.7	12.2	5.3	4.8	10.6	12.3
	>60 – <= 120	129	1.6	15.5	6.4	5.5	13.4	16.7
	>120 – <= 160	145	1.1	14.1	4.1	3.9	9.8	9.5
	160<	138	1.4	13.0	4.9	3.2	10.6	8.4
LongTerm ^&^	no	653	1.6	14.1	5.8	4.5	11.3	12.2
	yes	139	1.2	12.8	4.2	4.1	10.7	10.8
Transfer to NH ^§^	from home	270	1.5	13.0	4.9	4.3	10.8	11.2
	from hospital	289	1.4	15.4	5.7	4.9	11.7	13.5
Place of death	in NH	234	2.0	13.9	7.0	4.8	12.8	14.4
	in hospital	229	1.8	12.9	6.1	10.0	10.1	21.7

Total		792	1.4	13.5	5.0	4.4	11.1	11.9

^$ ^time of care required regarding basic activities of daily living

^&^Participants with NH stay for 1 year

^§ ^559 Participants from 2 of the largest health insurance plans

^%^Number of participants at time point of death

**Table 3:** Hospital admissions, sum of person-years in hospital and total costs during the last months of life according to main diagnosis of hospital stay

Main diagnosis for hospital stay (ICD-9/ICD-10)	Hospital admissions N (%)	Person-years in hospital PY (%)	costs € (%)
Infections (A00–B99, J10–J18, L00–L08, N30, N39/001–139, 480–487, 595, 599, 680–686)	104 (12.2)	3.5 (12.6)	299,701 (12.9)
- among these: Influenza and Pneumonia (J10–J18/480–487)	62 (7.3)	2.1 (7.5)	182,909 (7.9)
Cancer (C00–D48/140–199, 210–239)	52 (6.1)	1.5 (5.5)	134,942 (5.8)
Hormone-, nutrition-, metabolic diseases (E00–E90, N18–N19/240–279, 585–586)	62 (7.3)	2.3 (8.4)	175,235 (7.5)
Psychiatric Diseases (F00–F99/290–319)	47 (5.5)	3.2 (11.4)	215,685 (9.3)
- among these: Dementia (F00–F03/290)	10 (1.2)	0.7 (2.5)	54,234 (2.3)
Diseases of the nervous and sensory system (G00–G44, G47–G99/320–359)	25 (2.9)	0.9 (3.1)	82,029 (3.5)
Cerebrovascular diseases (G45–G46, I60–I69/430–438)	53 (6.2)	1.9 (6.7)	149,589 (6.4)
Cardiovascular diseases (D50–D89, I00–I59, I70–I99/200–208, 280–289, 390–429, 439–459)	146 (17.2)	4.3 (15.6)	362,387 (15.6)
- among these: Congestive heart failure (I50/428)	52 (6.1)	1.7 (6.0)	141,533 (6.1)
Diseases of the musculoskeletal system (M00–M99/710–739)	4 (0.5)	0.1 (0.3)	6,408 (0.3)
Injuries and poisoning (R55, S00–T98/780, 800–999)	78 (9.2)	2.3 (8.3)	241,059 (10.4)
- among these: Proximal Femur fractures (S72/820)	32 (3.8)	1.2 (4.4)	159,325 (6.9)
Diseases of the respiratory system (J00–J09, J19–-J99/460–479, 488–519)	79 (9.3)	2.1 (7.6)	184,416 (7.9)
Diseases of the digestive system (K00–K93/520–579)	93 (11.0)	2.8 (10.0)	233,321 (10.0)
Other diseases	106 (12.5)	2.9 (10.5)	240,124 (10.3)
Total*	849 (100.0)	27.7 (100.0)	2,324,896 €

*333 study participants without hospital admission between NHA and death

PY: Person years
